# Evaluating potential overuse of surveillance care in cancer survivors

**DOI:** 10.1002/cam4.5346

**Published:** 2022-11-11

**Authors:** Jennifer Y. Sheng, Claire F. Snyder, Katherine C. Smith, Jennifer DeSanto, Nancy Mayonado, Susan Rall, Sharon White, Amanda L. Blackford, Fabian M. Johnston, Robert L. Joyner, Joan Mischtschuk, Kimberly S. Peairs, Elissa Thorner, Phuoc T. Tran, Antonio C. Wolff, Youngjee Choi

**Affiliations:** ^1^ Johns Hopkins University School of Medicine Baltimore Maryland USA; ^2^ Johns Hopkins Sidney Kimmel Comprehensive Cancer Center Baltimore Maryland USA; ^3^ Johns Hopkins Bloomberg School of Public Health Baltimore Maryland USA; ^4^ TidalHealth Richard A. Henson Research Institute Salisbury Maryland USA

**Keywords:** breast cancer, clinical cancer research, clinical observations, colorectal cancer, prostate cancer

## Abstract

**Background:**

Survivorship care plans (SCPs) communicate cancer‐related information from oncology providers to patients and primary care providers. SCPs may limit overuse testing by specifying necessary follow‐up care. From a randomized, controlled trial of SCP delivery, we examined whether cancer‐related tests not specified in SCPs, but conducted after SCP receipt, were appropriate or consistent with overuse.

**Methods:**

Survivors of breast, colorectal, or prostate cancer treated at urban‐academic or rural‐community health systems were randomized to one of three SCP delivery arms. Tests during 18 months after SCP receipt were classified as consistent with overuse if they were (1) not included in SCPs and (2) on a guideline‐based predetermined list of “not recommended surveillance.” After chart abstraction, physicians performed review and adjudication of potential overuse. Descriptive analyses were conducted of tests consistent with overuse. Negative binomial regression models determined if testing consistent with overuse differed across study arms.

**Results:**

Among 316 patients (137 breast, 67 colorectal, 112 prostate), 140 individual tests were identified as potential overuse. Upon review, 98 were deemed to be consistent with overuse: 78 tumor markers and 20 imaging tests. The majority of overuse testing was breast cancer‐related (95%). Across sites, 27 patients (9%) received ≥1 test consistent with overuse; most were breast cancer patients (22/27). Exploratory analyses of overuse test frequency by study arm showed no significant difference.

**Conclusions:**

This analysis identified practice patterns consistent with overuse of surveillance testing and can inform efforts to improve guideline‐concordant care. Future interventions may include individual practice patterns and provider education.

## Graphical abstract

Cancer survivors at both academic and community cancer centers experienced overuse of surveillance testing. This analysis identified practice patterns consistent with overuse of surveillance patterns and can inform efforts to improve guideline‐concordant care.

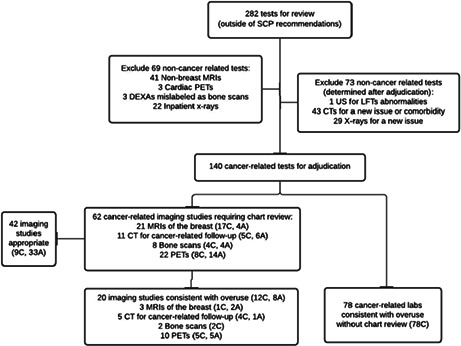

## 1 INTRODUCTION

With the aging population and advances in early cancer detection and treatment, the number of cancer survivors now exceeds 16 million in the United States.^1^ Clinical guidelines and survivorship care plans (SCPs), which outline posttreatment follow‐up recommendations, have been proposed to promote appropriate cancer surveillance for these patients.^2^ However, delivering high‐quality follow‐up care after cancer treatment for this population is challenging.^3^ One factor that contributes to the challenge of providing quality survivorship care is determining and delivering appropriate surveillance measures, which includes preventing both underuse and overuse. While many population‐based studies of breast, colorectal, and prostate cancer survivors have already demonstrated underuse in surveillance care, there may also be substantial overuse relative to guideline recommendations.^4^, ^5^, ^6^Surveillance patterns may be driven by provider knowledge or beliefs. In a cross‐sectional survey of providers, 84% of primary care providers and 72% of oncologists had beliefs consistent with blood test overuse, and 50% of primary care physicians and 27% of oncologists reported beliefs consistent with imaging test overuse.^7^ Few studies, however, have evaluated the appropriateness of individual surveillance tests received by patients, and studies conducted to date have generally not assessed the documentation of ordering providers in the medical record to determine whether tests were clinically indicated.^8^, ^9^, ^10^, ^11^ This study adds to the literature by reviewing the documentation in the medical records to evaluate whether surveillance tests were conducted for a reason or whether they are consistent with overuse.

Surveillance patterns are likely influenced by factors at multiple levels, including the health system, patient, and provider levels. From a randomized, controlled trial of SCP delivery at both an urban‐academic and rural‐community cancer center, we previously determined how SCP surveillance recommendations aligned with national guidelines for breast, colorectal, and prostate cancer survivors and found gaps related to follow‐up recommendations.^12^, ^13^, ^14^ The rationale for the study arms (mailed, delivered during a single visit, delivered during a visit with a 6‐month follow‐up visit) was for the primary analysis, which was receipt of care as recommended in SCPs. In the study's primary analysis of adherence to SCP‐recommended care, we found no differences by study arm. In this pre‐specified exploratory analysis, we examined delivery of tests that are not recommended for routine surveillance, and adjudicated whether such tests completed in the 18‐month period after SCP receipt were appropriate (i.e., done for cause) or consistent with overuse. This analysis was conducted to further explore potentially avoidable health care utilization that was cancer related.

## 2 METHODS

### 2.1 Study population

Patients recruited in this study were survivors of newly diagnosed or recurrent Stages I‐III breast, colorectal, or prostate cancer who were being treated at one of the two sites: (1) an urban, academic tertiary medical center with multiple oncology programs based on disease site and (2) a rural, regional community medical center with a single general oncology practice. Patients across the three arms of the overall study (mailed SCP or SCP delivered in the context of 1 or 2 visits) received an SCP within 6 months of treatment completion. Study SCP templates were adapted from the American Society of Clinical Oncology templates,^15^ and included a section for recommended surveillance testing. SCPs were completed by a member of the clinical team. While team members completing the SCPs were provided information based on the current American Society of Clinical Oncology guidelines to inform their recommendations, the provider completing the SCP could use their discretion and tailor their surveillance recommendations for each individual. The study was approved by the Institutional Review Board at XXX (removed for review) and registered on clinicaltrials.gov as XXX (removed for review).

### 2.2 Data collection

**FIGURE 1:**
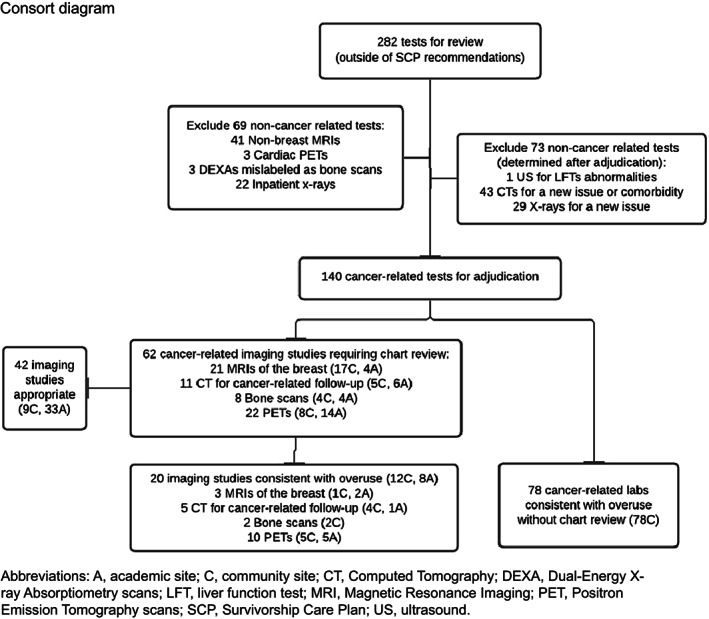
Consort diagram

For each patient, we collated the laboratory and imaging tests performed during the 18 months after SCP receipt that were predetermined in the study protocol as “not recommended surveillance” (Table 1). We collected information including the type of test, anatomic site, reason for test, and ordering provider. We initially included for review all tests received over 18 months if the test type was not included in the SCP, or the total number of tests performed exceeded the maximum specified number for the 18‐month period in the SCP. Non‐cancer‐related tests (e.g., non‐breast MRIs, cardiac PET, DEXA mislabeled as bone scan) and tests ordered during hospitalization (e.g., inpatient x‐rays) were excluded **(**Figure 1 **)**.

**TABLE 1:** Surveillance testing recommendations for Stages I–III Cancer predetermined for the study protocol^a^

Cancer type	Breast	Colorectal	Prostate
Recommended surveillance	Annually: Mammography (excluding women with bilateral mastectomy)	Every 3–6 months: CEA Annually: CT chest/abdomen/pelvis Colonoscopy (can be done at year 3 if preoperative full colonoscopy normal)	Every 6–12 months: PSA
Not recommended surveillance (assumes normal H&P and normal recommended surveillance)	Imaging Breast MRI PET/CT CT chest/abdomen/pelvis Liver ultrasound Bone scan Blood tests Tumor markers	Imaging Chest x‐ray PET/CT	Imaging PET/CT Bone scan

Abbreviations: CEA, carcinoembryonic antigen; CT, computed tomography; MRI, magnetic resonance imaging; PET, positron emission tomography scans; PSA, prostate specific antigen.

^a^ Adapted from 2013 National Comprehensive Cancer Network and American Society of Clinical Oncology guidelines.

Initially, one research team member abstracted the laboratory/imaging testing from the medical record, and a second research team member reviewed the abstraction. Subsequently, two physicians (one general internist, one medical oncologist) reviewed the abstracted ordering provider's documentation in the medical record for tests ordered outside SCP recommendations. Tests determined to have been ordered for non‐cancer ‐related work were excluded. If a test was not advised under any circumstance for surveillance from national guidelines (e.g., tumor markers in breast cancer), it was designated as consistent with overuse. In instances when it was unclear why testing was ordered from the information abstracted by research staff members, a medical oncologist performed additional chart review to adjudicate whether the test was consistent with overuse. A second physician reviewer who was blinded to the first reviewer's assessments separately adjudicated 10% of the tests to ensure agreement. Of the six tests reviewed, there was concordance on five of the tests, and agreement on the remaining test after additional discussion so we proceeded with single adjudication,

The updated American College of Radiology recommendations mentions use of breast MRI for women with a personal history of breast cancer, either dense tissue or who were diagnosed before the age of 50.^16^ To allow for a reasonable breadth of indications when determining appropriate use versus overuse, we included these additional guideline parameters as clinical indications for breast MRI.

### 2.3 Statistical analysis

To be included in this analysis, patients had to receive an SCP and complete 18 months of follow‐up. We performed a descriptive analysis of tests ordered outside of SCPs, including frequency and percentage of tests consistent with overuse, and patients with testing consistent with overuse. Negative binomial regression was used to determine if the rate of testing consistent with overuse differed across the three study arms of the SCP delivery method. There were no hypotheses for these exploratory analyses.

## 3 RESULTS

Among 316 patients in the study, a total of 282 tests were ordered that were not indicated in SCP recommendations (Figure 1). With initial review, 69 non‐cancer‐related tests were excluded; these included 41 non‐breast magnetic resonance imaging (MRI) tests, three cardiac positron emission tomography (PET) scans, three dual‐energy x‐ray absorptiometry (DEXA) scans that had been mislabeled as bone scans (by initial coding from the study team), and 22 inpatient x‐rays. The inpatient x‐rays were excluded as they were performed for workup of non‐cancer‐related etiologies of issues, and none of them yielded results related to cancer. An additional 73 tests were excluded after they were adjudicated to be non‐cancer‐related tests; these included an abdominal ultrasound, 43 computed tomography (CT) scans for a new issue or comorbidity, and 29 x‐rays for a new issue. Ultimately, 140 cancer‐related tests were identified and then adjudicated for possible overuse; 78 tumor marker tests were determined to be consistent with overuse without chart review, while 62 imaging tests required additional chart review. Of the 62 cancer‐related imaging studies, 20 were deemed consistent with overuse (12 community site, 8 academic site), and 42 were deemed appropriate (9 community site, 33 academic site) **(**Figure 1 **)**. Documentation in the medical record for appropriate testing included new concerning clinical or radiographic findings, new symptoms concerning for cancer recurrence, breast reconstruction after surgery, and follow‐up for high‐risk breast cancer surveillance (Table 2). Provider documentation in the medical record for lab and imaging testing consistent with overuse included: routine cancer surveillance, chronic symptoms, elevated tumor markers (initial order was not consistent with guidelines), and not fulfilling high‐risk breast cancer surveillance criteria. The most commonly ordered imaging consistent with overuse included PET scans (10/20, 50%), CTs (5/20, 25%), and breast MRIs (3/20, 15%). Provider documentation in the medical record for PET scans consistent with overuse included: cancer surveillance, initial workup for a new symptom, and elevated breast tumor markers (which providers are advised against checking).

**TABLE 2:** Results of adjudication by chart review across both sites (*N* = 62)

Cancer type	Testing (*n*)	Appropriate (*n*, %)	Consistent with overuse (*n*, %)
Breast	Breast MRI (21)	Age < 50 at diagnosis or dense/heterogeneous tissue (15, 71%) Recommended follow‐up (2, 10%) Workup for symptoms (1, 5%)	Does not fulfill ACR guidelines for high risk screening (3, 15%)
	CT (11)	For reconstruction (6, 55%)	Symptoms (1, 9%) Surveillance (4, 36%)
	PET (11)	Assess response to radiation therapy (1, 9%) Follow‐up of previous radiographic abnormality (5, 45%)	Symptoms (1, 9%) Elevated tumor markers (3, 27%) Surveillance (1, 9%)
	Bone scan (8)	Work up for symptoms (5, 63%) Follow‐up of previous radiographic abnormality (1, 13%)	Surveillance (1, 13%) Chronic symptoms (1, 13%)
Colorectal	PET (9)	Follow‐up of previous radiographic abnormality (3, 33%) Elevated CEA or lymphadenopathy (2, 22%)	Surveillance (4, 44%)
Prostate	PET (2)	Elevated PSA and bone lesion (1, 50%)	Surveillance (1, 50%)

*Note*: Appropriate rationales for testing included new concerning clinical or radiographic findings, new symptoms concerning for cancer recurrence (e.g., new and persistent back pain), breast reconstruction after surgery, and follow‐up for high‐risk breast cancer surveillance. Rationales for testing consistent with overuse included routine cancer surveillance, symptoms (e.g., CT for initial infectious workup), elevated tumor markers (initial order not consistent with guidelines), and not fulfilling high‐risk breast cancer surveillance criteria.

Abbreviations: ACR, American College of Radiology; CEA, carcinoembryonic antigen; CT, computed tomography; MRI, magnetic resonance imaging; PET, positron emission tomography; PSA, prostate‐specific antigen.

The majority of testing consistent with overuse was related to tumor markers (78/98, 80%) and subsequent imaging that was ordered to evaluate abnormal tumor marker values (3/98, 3%). Additionally, the majority of testing consistent with overuse was breast cancer‐related testing across recruitment sites (93/98, 95%). There was no significant difference in the incidence of overuse tests between SCP delivery study arms (*p* = 0.65).

### 3.1 Overuse testing by recruitment site and tumor type

Of the 140 cancer‐related tests ordered that were not recommended on SCPs (including tumor markers), 91% (90/99) at the community site were consistent with overuse and 20% (8/41) at the academic site were consistent with overuse. The majority of testing consistent with overuse at the community site (78/90, 87%) was related to laboratory testing. Excluding laboratory testing, 57% (12/21) of imaging tests performed at the community site were consistent with overuse. In contrast, the academic site performed no lab testing consistent with overuse, although 20% (8/41) of cancer‐related imaging at the academic site was consistent with overuse. By cancer type, the rate of testing consistent with overuse was most common for breast cancer patients at the community site (18/65, 28%) and for colorectal patients at the academic site (4/46, 9%) (Table 3).

**TABLE 3:** Frequency and percentage of cancer survivors with overuse by study site and cancer type

	Academic site				Community site				All sites
	Breast *N* = 72	Colorectal *N* = 46	Prostate *N* = 46	All cancer types *N* = 164	Breast *N* = 65	Colorectal *N* = 21	Prostate *N* = 66	All cancer types *N* = 152	All cancer types *N* = 316
Any overuse—no. (%)									
None	68 (94%)	42 (91%)	46 (100%)	156 (95%)	47 (72%)	21 (100%)	65 (98%)	133 (88%)	289 (91%)
Some	4 (6%)	4 (9%)	0 (0%)	8 (5%)	18 (28%)	0 (0%)	1 (2%)	19 (13%)	27 (9%)

### 3.2 Overuse testing at the patient level

From the 316 patients in the study (137 breast, 67 colorectal, 112 prostate), 111 patients had tests that were identified for review as potential overuse. After excluding non‐cancer‐related tests, 53 patients had at least one cancer‐related test that needed adjudication for overuse. Some patients had multiple tests ordered (e.g., serial tumor markers, tumor markers and CT, tumor markers and PET). Ultimately, 27 patients were determined to have at least one test consistent with overuse. Among the 27 patients with any testing consistent with overuse, 22 were breast cancer patients. A total of 16 breast cancer patients at the community site accounted for all 78 tumor marker tests consistent with overuse. By cancer type, breast cancer patients most frequently had at least one test consistent with overuse (22/137, 16%) (Table 3). Overall, the least amount of testing consistent with overuse occurred in prostate cancer patients (1/112, <1%).

## 4 DISCUSSION

We assessed the appropriateness of cancer‐related testing not recommended in the SCPs of early‐stage breast, colorectal, and prostate cancer survivors enrolled in a trial of SCP delivery. We accounted for provider reasons for test ordering based on documentation in the medical record, although surveying providers would be required to understand their full rationale. We found that (1) testing consistent with overuse mainly consisted of tumor markers for breast cancer and (2) a substantial minority (approximately one‐third) of imaging studies ordered outside of SCP recommendations were consistent with overuse.

Tumor markers accounted for the majority of testing consistent with overuse, occurred exclusively at the community site, and were all breast cancer‐related testing. While the 2000 National Comprehensive Cancer Network guidelines included serum tumor markers for breast cancer (CEA, CA 15–3, and CA 27.29), all societal guidelines since then have recommended against this testing.^17^ The medical record did not document reasoning for tumor markers orders. Drivers for ordering these tests are multifactorial and could include: concerning cancer characteristics (higher stage), patient directed care (more anxious patients seeking this testing), lack of knowledge among providers (including older practitioners whose practices are outdated and align with older guidelines), health systems, and regional variation in practice patterns. While other studies have assessed overordering of tumor markers, none directly compared these between a community and academic site.^18^, ^19^ They do evaluate provider motivations related to overuse (e.g., patients displaying nonspecific signs/symptoms, avoidance of a more expensive diagnostic imaging procedure) and patient features (e.g., stage of cancer). Another prior study assessing oncologists' beliefs about surveillance found the following were associated with overuse testing: older age, international medical graduate status, lower confidence in knowledge, and greater perceptions of ambiguity. ^7^ In the same study, primary care physicians with greater overuse were more often associated with health system features, such as a smaller practice size, lower patient volume, and practice ownership. While we were unable to explore the factors behind testing decisions in our analysis, future studies examining why tumor markers get ordered in early‐stage breast cancer survivors would help target strategies to limit low‐value testing discordant with guidelines.

Two‐thirds of imaging studies ordered, but not recommended on the SCP, were determined to be appropriate after adjudication. These tests included PET or bone scans to further evaluate a new concerning symptom, radiographic abnormality, or elevated guideline‐recommended tumor marker (i.e., PSA, CEA), and CTs ordered for breast reconstruction planning. With breast MRIs, we observed that the majority had indications for testing based on the American College of Radiology recommendations, which go beyond other national guidelines (e.g., American Society of Clinical Oncology, National Comprehensive Cancer Network). These findings bring attention to the variation in national cancer guidelines that may lead to a lack of clarity for clinicians trying to order appropriate surveillance testing. Different societies should consider collaborating to define individuals who may benefit most from breast MRI surveillance, using risk‐stratification to determine who is high risk based on the literature.^20^ Risk stratification could consider factors such as increased familial risk for the development of breast cancer or prior medical history (use of radiation therapy, history of breast cancer, and breast density).^16^, ^21^, ^22^ The remaining one‐third of imaging studies were consistent with overuse, including PET scans ordered for “cancer surveillance,” PET scans to follow‐up inappropriately ordered tumor markers (i.e., in breast cancer), and various advanced imaging studies that replaced an initial routine workup for a symptom. Similar to breast cancer tumor marker testing, we were unable to determine what factors led to imaging tests consistent with overuse. There may be other factors at play that drove those decisions that are not well captured in the medical record. A previous study by Enright et al. examined imaging studies ordered within the first 2 years after curative treatment in breast cancer patients. The rates of advanced imaging tests (defined as CT, bone scan, and PET) increased with older age, higher disease stage, comorbidity, chemotherapy exposure, and prior staging investigations.^6^ Unlike our study, they did not assess provider documentation in the medical record and whether these imaging tests were consistent with overuse. Further studies to identify and intervene upon factors associated with oversurveillance imaging will be important to decrease unnecessary imaging for cancer survivors.

This study had several strengths and limitations. We examined the appropriateness of cancer‐related testing by accounting for provider documentation in the medical record for any given test. While we know of three other studies that have assessed overuse of surveillance testing in a similar population of cancer survivors,^4^, ^5^, ^6^ their methods precluded assessment of the reason for testing to determine appropriateness. While we have gone further than prior studies in abstracting provider documentation in the medical record, we were not able to evaluate individual provider or patient factors that may impact ordering of tests consistent with overuse. Evaluation of these factors could be important in better understanding when testing outside of national recommendations is more likely to occur, and point to possible intervention opportunities at the patient provider or system level. In addition, there may be other factors that drove provider decisions that were not fully captured in the medical record. In our study, we also evaluated testing at an urban and rural site, to capture patterns that might vary based on practice location. To improve generalizability, similar studies looking at practice patterns across more institutions would be helpful. Additionally, future studies would be strengthened if there are methods to assess reasons for testing or visits with a larger number of participants. However, incorporating methodology (such as that utilized in our study) that allows for detailed and comprehensive consideration of documentation in the medical record for orders and procedures may be challenging to perform with larger numbers.

In conclusion, our study found patterns of surveillance testing reflective of overuse relative to national guideline recommendations among breast, colorectal, and prostate cancer patients. In particular, tumor markers consistent with overuse were commonly ordered in early‐stage breast cancer survivors at the community site, and PET scans were used for routine surveillance across cancer types and both sites. Future larger studies should evaluate whether overuse testing is more prevalent in certain subgroups. Furthermore, these results suggest modifiable targets for reducing surveillance test overuse, and further studies using qualitative and mixed‐methods approaches should explore individual provider practice patterns and drivers of these testing patterns.

## AUTHOR CONTRIBUTIONS

**Jennifer Y. Sheng:** Conceptualization (equal); formal analysis (equal); methodology (equal); writing – original draft (equal); writing – review and editing (equal). **Claire F. Snyder:** Conceptualization (equal); data curation (equal); formal analysis (equal); methodology (equal); writing – original draft (equal); writing – review and editing (equal). **Katherine C. Smith:** Conceptualization (equal); data curation (equal); formal analysis (equal); methodology (equal); writing – original draft (equal); writing – review and editing (equal). **Jennifer DeSanto:** Data curation (equal); writing – original draft (equal); writing – review and editing (equal). **Nancy Mayonado:** Data curation (equal); writing – original draft (equal); writing – review and editing (equal). **Susan Rall:** Data curation (equal); writing – original draft (equal); writing – review and editing (equal). **Sharon White:** Data curation (equal); writing – original draft (equal); writing – review and editing (equal). **Amanda L. Blackford:** Conceptualization (equal); formal analysis (equal); methodology (equal); writing – original draft (equal); writing – review and editing (equal). **Fabian M. Johnston:** Data curation (equal); formal analysis (equal); writing – original draft (equal); writing – review and editing (equal). **Robert L. Joyner:** Data curation (equal); formal analysis (equal); writing – original draft (equal); writing – review and editing (equal). **Joan Mischtschuk:** Data curation (equal); writing – original draft (equal); writing – review and editing (equal). **Kimberly S. Peairs:** Conceptualization (equal); writing – original draft (equal); writing – review and editing (equal). **Elissa Thorner:** Conceptualization (equal); formal analysis (equal); methodology (equal); writing – original draft (equal); writing – review and editing (equal). **Phuoc T. Tran:** Data curation (equal); formal analysis (equal); writing – original draft (equal); writing – review and editing (equal). **Antonio C. Wolff:** Conceptualization (equal); writing – original draft (equal); writing – review and editing (equal). **Youngjee Choi:** Conceptualization (equal); data curation (equal); formal analysis (equal); methodology (equal); writing – original draft (equal); writing – review and editing (equal).

## FUNDING INFORMATION

This study was funded through a PCORI Award (IHS‐1409‐22534). The Sidney Kimmel Comprehensive Cancer Center at Johns Hopkins receives funding from the National Cancer Institute (P30CA006973).

## CONFLICT OF INTEREST

All authors received Patient‐Centered Outcomes Research Institute (PCORI) research funding through their institution. Dr. Sheng has received Pfizer research funding through her institution. Dr. Snyder has received personal royalties for section authorship in up‐to‐date, personal consulting fees from Janssen (via Health Outcomes Solutions), and research funding from Genentech and Pfizer through her institution. Dr. Johnston has received NIH grant 1R01CA252101‐01A1. Dr. Joyner serves as a non‐paid board member for the National Board for Respiratory Care, Salisbury University Alumni, and Salisbury University College of Health and Human Sciences. Dr. Tran received research funding from Astellas Pharm, Bayer Healthcare, and RefleXion Medial Inc; personal fees from Consulting from RefleXion, Janssen‐Taris Biomedical, Myovant and AstraZeneca outside the submitted work; and has a patent 9,114,158 ‐ Compounds and Methods of Use in Ablative Radiotherapy licensed to Natsar Pharm. Dr. Peairs and Dr. Choi have received research funding from a Merck Foundation Grant through their institution.

## ETHICS STATEMENT

The study complies with the Declaration of Helsinki and was approved by the Institutional Review Board at the Johns Hopkins School of Medicine. All patients provided their written informed consent.

## DISCLAIMERS

The statements in this publication are solely the responsibility of the authors and do not necessarily represent the views of PCORI, its Board of Governors or Methodology Committee.

## Data Availability

The datasets generated and analyzed during the current study are available from the corresponding author on request
